# The initial and ongoing effect of the COVID-19 pandemic on the reach and impact of a US state tobacco quitline

**DOI:** 10.18332/tpc/203869

**Published:** 2025-05-23

**Authors:** Lawrence C. An, Karen S. Brown, Allison K. C. Furgal, Mohammed A. Saqib, Farid J. Shamo

**Affiliations:** 1 Department of Internal Medicine, Division of General Medicine, University of Michigan, Ann Arbor, United States; 2 Tobacco Control Program, Michigan Department of Health and Human Services, Lansing, United States; 3 Department of Biostatistics, University of Michigan, Ann Arbor, United States

**Keywords:** impact, tobacco cessation, nicotine replacement therapy, COVID-19, reach, tobacco quitline

## Abstract

**INTRODUCTION:**

This study examines the long-term effect of the COVID-19 pandemic on the reach and impact of one US state tobacco quitline while taking into account quitline offers of free nicotine replacement therapy (NRT).

**METHODS:**

This is a pre-post analysis from January 2017 through June 2023 of the reach and impact of Michigan’s tobacco quitline after the start of the COVID-19 pandemic. We assess quitline reach (number of callers per month), effectiveness (self-reported 30-day abstinence at 6 months), and impact (number of new ex-tobacco users per month). We examine the main effects and interactions between pandemic status (i.e. pre vs post March 2020) and quitline offers of free NRT.

**RESULTS:**

The COVID-19 pandemic had a persistent negative effect on quitline reach (p=0.002) and impact (p<0.001). Abstinence rates decreased transiently during the first year of the pandemic. Offering free NRT had a positive effect on quitline reach (p<0.001) and impact (p<0.001) before and after the start of the pandemic. For quitline reach, we found a significant interaction between pandemic and free NRT effects with a substantial decrease in the mean number of callers per month after the pandemic during months when free NRT is being offered (750; 95% CI: 545–1033, pre-pandemic vs 302; 95% CI: 233–392, post-pandemic) compared to months when free NRT is not being offered (247; 95% CI: 187–327, pre-pandemic vs 159; 95% CI: 114–221, post-pandemic).

**CONCLUSIONS:**

There is a critical need to assess and address the ongoing effects of the COVID-19 pandemic on tobacco quitline reach and impact.

## INTRODUCTION

The widespread adoption of tobacco quitlines represents a major advance in tobacco control^1^. Early in the COVID-19 pandemic, concerns were raised regarding reduced quitline engagement^2^. The North American Quitline Consortium (NAQC) reported a 27% decrease in US quitline call volume during 2020^3^. The purpose of this short report is to present the reach (i.e. callers per month) and impact (i.e. successful quitters per month) of the Michigan state tobacco quitline before and after the start of the COVID-19 pandemic. Because offering free nicotine replacement therapy (NRT) is a recognized strategy for increasing quitline reach and impact^4-6^, we examine the pandemic’s effects on quitline reach and impact during periods of time when free NRT was or was not being offered.

## METHODS

### Design and setting

This is a pre-post analysis (January 2017 – June 2023) of the reach and impact of Michigan’s state tobacco quitline (Michigan Tobacco Quitlink) after the start of the COVID-19 pandemic. The first cases of COVID-19 were reported in Michigan in March of 2020.

Michigan Tobacco Quitlink services are provided by National Jewish Health. Quitlink employs an evidence-based four-call protocol. Calls are scheduled in a relapse-sensitive fashion and address smoking and quitting history, motivations to quit, overcoming barriers to quitting, and maintenance of abstinence^7^. The Michigan Tobacco Quitlink offers 2 weeks of free NRT to all callers for typically 6–7 months out of each year as part of annual quitline promotional efforts.

### Measures

We assessed quitline reach in terms of the number of callers each month. Callers are asked if they are willing to complete follow-up evaluation surveys. We assessed self-reported 30-day abstinence rates at 6 months among respondents to a telephone survey of this volunteer sample. Abstinence rates are only available in aggregate by fiscal year (July– June). We assessed quitline impact by multiplying the specific monthly call volume by the appropriate annual abstinence rate to estimate the number of new ex-tobacco users among quitline callers each month.

### Statistical analysis

We analyzed quitline reach and impact using negative binomial regression models with log transformation of model outcomes. For both models, the independent predictor variables were indicators specifying: 1) pre- versus post-pandemic status (i.e. before vs after March 2020), and 2) whether free NRT was offered that month. Time in months was included as a continuous variable. We examined the main effects and interactions between pandemic status and the offer of free NRT. Model outputs are back transformed to present the estimated number (with 95% confidence intervals) of callers and new ex-smokers per month. Analyses were performed with R version 4.4.0.

### Ethical considerations

This study was reviewed by the University of Michigan’s IRB-Med and determined to be a nonregulated quality assurance activity (HUM00264664).

## RESULTS

**Figure 1:**
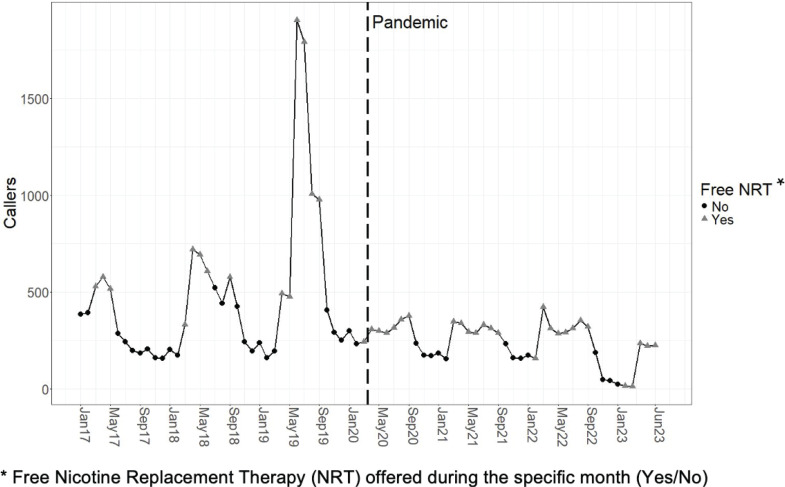
Michigan Tobacco Quitlink monthly call volume before and after the start of the COVID-19 pandemic (January 2017–June 2023)

Overall, quitline call volume decreased 49% from an average of 465 (SD=394) calls per month before the pandemic to 237 (SD=105) calls per month after the pandemic. Monthly quitline call volume is shown in Figure 1. A comparison of caller demographics shows modest increases in caller age, the proportion of non-White callers, and callers with any health insurance (Supplementary file Table 1).

The main effects model examining the number of callers per month showed a significant negative pandemic effect (p=0.002) and a significant positive free NRT effect (p<0.001) with no significant overall time trend (p=0.47). The interaction model showed a significant interaction between pandemic and free NRT effects (p=0.047). Before the start of the pandemic, the estimated mean number of callers per month was 247 (95% CI: 187–327) without free NRT and 750 (95% CI: 545–1033) when free NRT was being offered. After the start of the pandemic, the estimated mean number of callers per month is 159 (95% CI: 114–221) without free NRT and 302 (95% CI: 233–392) when free NRT is being offered. The effect of the interaction is evident in that there is a substantial decrease in the number of callers per month (with no overlap in the confidence intervals) after the pandemic during months when free NRT is being offered, compared to months when free NRT is not being offered.

The 30-day abstinence rates at six months for Fiscal Year 2017 (FY2017) through Fiscal Year 2023 (FY2023) ranged from 22.9% to 29.2%. The abstinence rates by fiscal year are as follows: FY2017, 24.0% (95% CI: 21.1–26.8); FY2018, 28.0% (95% CI: 25.4–30.8); FY2019, 29.2% (95% CI: 26.3–31.4); FY2020, 28.6% (95% CI:26.1–31.2); FY2021, 22.9% (95% CI: 20.0–26.1); FY2022, 26.9% (95% CI: 23.7–30.1); FY2023, 27.0% (95% CI: 24.4–30.4). The abstinence rate for FY2022 (July 2020–June 2021), which largely corresponded to the first year of the COVID-19 pandemic, was the only year that was significantly lower than other years.

The main effects model examining the number of new ex-tobacco users per month showed a significant negative pandemic effect (p<0.001) and a significant positive free NRT effect (p<0.001), with no significant overall time trend (p=0.93). In the interaction model, the interaction between pandemic status and free NRT effects (p=0.065) was not significant. Before the pandemic, the estimated mean number of new ex-tobacco users per month was 75 (95% CI: 57–98) without free NRT and 234 (95% CI: 164–302) when free NRT was being offered. After the pandemic, the estimated mean number of new ex-tobacco users per month is 37 (95% CI: 27–52) per month without free NRT and 73 (95% CI: 57–94) when free NRT is being offered. The estimated number of new ex-tobacco users is lower after the start of the pandemic in months when free NRT is and is not being offered (with no overlap in confidence intervals).

Full output from main effect and interaction models and details on the evaluation of abstinence rates are included in the Supplementary file.

## DISCUSSION

This analysis demonstrates a persistent decrease in the impact of a US state tobacco quitline after the start of the COVID-19 pandemic. The reduction in quitline impact appears to be largely driven by a decrease in program reach (i.e. call volume). Quitline effectiveness (i.e. abstinence rates) decreased transiently with a significant decrease only during the first year of the pandemic. This is the first analysis to report on potential interactions between pandemic effects and quitline offers of free NRT. Our analysis supports the well-recognized benefit of offering free NRT as part of quitline services while noting that the COVID-19 pandemic has partially limited these benefits, particularly in terms of quitline reach.

The decrease in call volume that we report is generally consistent, though somewhat larger than changes in US national quitline call volume. NACQ recently reported that deceases in call volume in 2020 largely persisted through 2021^8^. Separate analysis of the CDC’s *Tips from Former Smokers* media campaign shows a significant negative time trend in national quitline call volume through 2023, though this analysis did not explicitly assess for effects of the pandemic or offers of free NRT^9^.

It is important to consider our findings in the context of the broader impact of the COVID-19 pandemic on tobacco use and cessation behaviors. Data from the Behavioral Risk Factor Surveillance System (BRFSS) show a modest decrease in the prevalence of smoking early in the pandemic^10^. Additional work showed a decrease in the prevalence of past-year quit attempts among current smokers^11^. BRFSS data from Michigan are consistent with these overall trends showing a decrease in smoking prevalence (18.7%, 2019; 18.4%, 2020; 17.0%, 2021; 15.2%, 2022) and past-year quit attempts (59.1%, 2019; 56.2%, 2020; 57.1%, 2021; 52.5%, 2022) that could have contributed to the changes we observe in quitline reach and impact^12^.

The causes for decreased quitline engagement are not clear. Media expenditures promoting the Michigan Tobacco Quitlink were reduced in 2021 but have since exceeded pre-pandemic levels. Many population-level effects of the pandemic, such as worsening mental health, decreased health care access, lower engagement with preventive care, and loss of trust in healthcare workers and institutions, could have contributed to these findings^13-16^.

### Limitations

There are several limitations to this analysis. First, this is a pre-post study of one US state tobacco quitline. Results for other US state quitlines or quitlines globally could certainly vary. Second, abstinence rates for quitline callers are only available in aggregate by year. We are not able to determine abstinence rates separately for months when free NRT is being offered, and this could lead us to underestimate the abstinence effect and overall impact of offering free NRT. Finally, we cannot rule out the possibility that factors other than the COVID-19 pandemic could have contributed to our findings.

## CONCLUSIONS

This study highlights the impact of the COVID-19 pandemic on quitline reach and impact. In pandemics, quitlines should continue to offer free NRT but additional efforts are needed to restore and maximize the impact of this offer. Future work is needed to identify the range of pandemic impacts on different quitlines and factors that contribute to differential impact. Ideally, this work could lead to the formulation of best practices to address the effects of pandemics on quitlines.

## Supplementary Material



## Data Availability

The data supporting this research are available from the authors on reasonable request.
