# Supplementary material for: The initial and ongoing effect of the COVID-19 pandemic on the reach and impact of a US state tobacco quitline
Source: Tob Prev Cessat. 2025 May 23;11:10.18332/tpc/203869. doi: 10.18332/tpc/203869 (PMC12100942; doi:10.18332/tpc/203869)
Supplement: Supplementary file 1 [file TPC-11-26-s1.pdf]

Supplemental tables and figures

Supplemental Table 1. Demographic characteristics of Michigan Tobacco Quitlink callers before (January 2017-February 2020) and after (March 2020-June 2023) the start of the COVID-19 pandemic.

	**Pre-pandemic (January 2017-February 2020)**		**Post-pandemic (March 2020-June 2023)**		
**Caller Characteristics**	**N**	**Mean or %**	**N**	**Mean or %**	**p-value***
**Total calls**	17541		9174		
**Age**	17475		9113		
Mean age		53.4		55.4	p<0.001
18-24	429	2.45%	157	1.72%	p < 0.001
25-34	1707	9.77%	710	7.79%	
35-44	2253	12.89%	1079	11.84%	
45-64	9339	53.44%	4712	51.71%	
65+	3747	21.44%	2455	26.94P%	
**Gender**	17541		9174		
Male	6458	36.81%	3288	35.84%	p = 0.02
Female	10885	62.05%	5749	62.67%	
Other	198	1.13%	137	1.49%	
**Ethnicity**	17204		8963		
White	12245	71.18%	6067	67.69%	p < 0.001
Black	3494	20.31%	2162	24.12%	
Hispanic	194	1.13%	108	1.20%	
Other	371	2.16%	191	2.13%	
Multi-Racial	900	5.23%	435	4.85%	
**Health insurance**	17283		9010		
No Insurance	2506	14.50%	961	10.67%	p < 0.001
Any Insurance	14777	85.50%	8049	89.33%	

*t-test for comparision of means and chi-square test for comparision of proportions

Supplemental Table 2: Quit rate data

Fiscal Year	Months included in data	Survey Pool	Completed Survey	Response Rate	Quit Rate	Quit Rate 95% CI
FY 2017	Jan 2017-Jun 2017	4885	887	18%	24%	(21.1% - 26.8%)
FY 2018	Jul 2017-Jun 2018	3973	1070	27%	28%	(25.4% - 30.8%)
FY 2019	Jul 2018-Jun 2019	5337	1225	23%	29.2%	(26.3% – 31.4%)
FY 2020	Jul 2019-Jun 2020	5671	1288	23%	28.6%	(26.1% – 31.2%)
FY 2021	Jul 2020-Jun 2021	3087	780	25%	22.9%	(20.0% – 26.1%)
FY 2022	Jul 2021-Jun 2022	3540	734	21%	26.9%	(23.7% – 30.1%)
FY 2023	Jul 2022-Jun 2023	3379	861	25%	27%	(24.4% - 30.4%)

Supplemental Table 3: Callers model results

**Outcome: Callers**	**Main effects model**			**Interaction model**		
**Effect**	**Estimate**	**Std. Error**	**p-value**	**Estimate**	**Std. Error**	**p-value**
Intercept	5.62	0.14	<0.0001	5.51	0.14	<0.0001
Free NRT (ref: No Free NRT)	0.87	0.12	<0.0001	1.10	0.17	<0.0001
Pandemic (ref: Pre-Pandemic)	-0.71	0.23	0.0023	-0.44	0.26	0.0933
Month (continuous)	0.00	0.01	0.4734	0.00	0.00	0.3824
Free NRT-Pandemic interaction (ref: No Free NRT and Pre-Pandemic)	NA	NA	NA	-0.47	0.24	0.0472

Supplemental Table 4: Ex-tobacco users model results

**Outcome: Ex-tobacco users**	**Main effects model**			**Interaction model**		
**Effect**	**Estimate**	**Std. Error**	**p-value**	**Estimate**	**Std. Error**	**p-value**
Intercept	4.41	0.13	<0.0001	4.32	0.14	<0.0001
Free NRT (ref: No Free NRT)	0.88	0.12	<0.0001	1.09	0.16	<0.0001
Pandemic (ref: Pre-Pandemic)	-0.95	0.22	<0.0001	-0.70	0.25	0.006
Month (continuous)	0.00	0.00	0.9306	0.00	0.00	0.9408
Free NRT-Pandemic interaction (ref: No Free NRT and Pre-Pandemic)	NA	NA	NA	-0.42	0.23	0.0653

Supplemental Figure 1: Mean number of Callers with 95% confidence interval, by Pandemic and Free NRT, estimated from interaction model

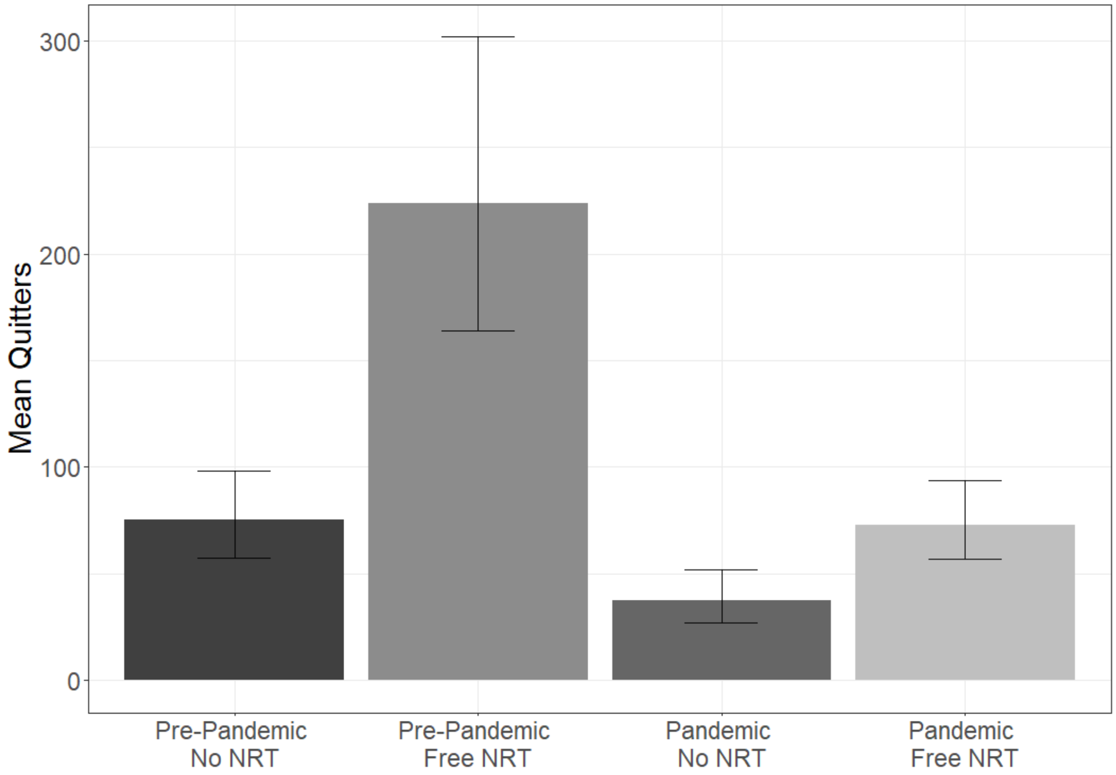

Supplemental Figure 2: Mean number of Ex-tobacco users with 95% confidence interval, by Pandemic and Free NRT, estimated from interaction model

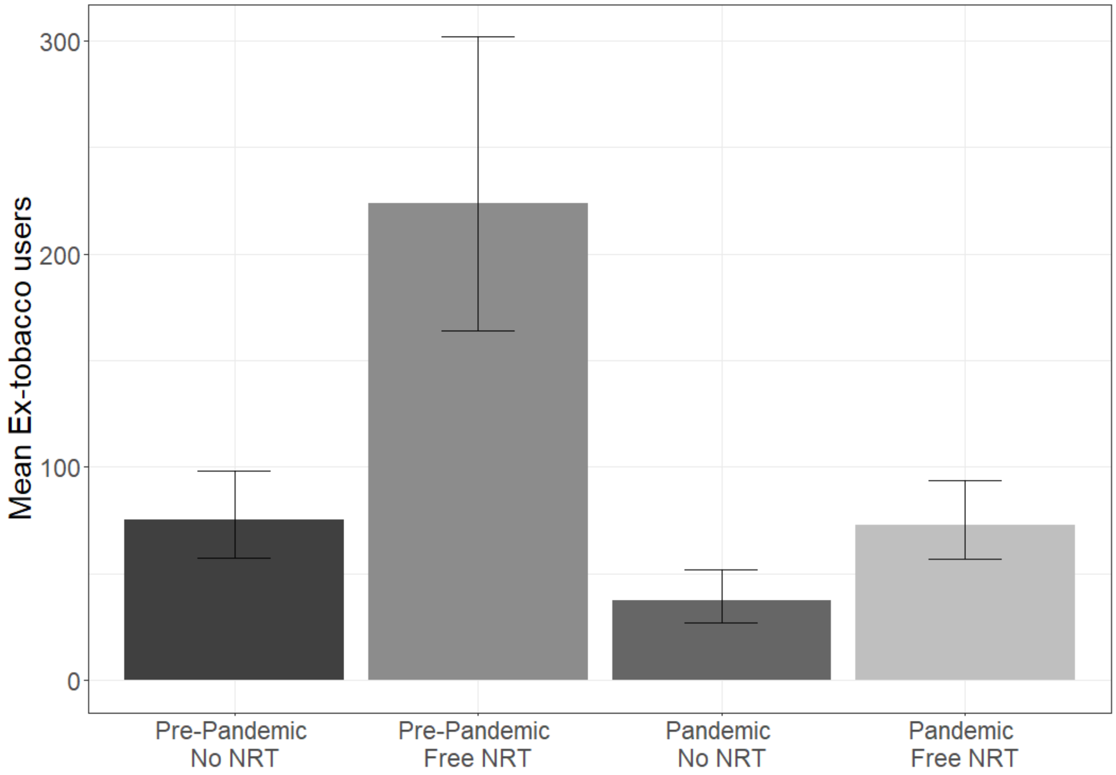

© 2025 An L.C. et al.
